# Strengthening Capacity for Implementation Research Amid COVID-19 Pandemic: Learnings From the Global Alliance for Chronic Diseases Implementation Science School

**DOI:** 10.3389/ijph.2022.1604944

**Published:** 2022-08-09

**Authors:** Zahra Aziz, Tilahun Haregu, Catherine Kyobutungi, Lijing Yan, Vilma Irazola, Pilvikki Absetz, Isobel Bandurek, Morven Roberts, Rajesh Vedanthan, Sheree Folkes, Yingting Cao, Yu Wen, Myo Nyein Aung, Katrien Danhieux, Allissa Desloge, Brian Oldenburg

**Affiliations:** ^1^ School of Psychological Sciences, Monash University, Melbourne, VIC, Australia; ^2^ Baker Heart and Diabetes Institute, Melbourne, VIC, Australia; ^3^ African Population and Health Research Center, Nairobi, Kenya; ^4^ Duke Kunshan University, Kunshan, China; ^5^ Instituto de Efectividad Clinicay Sanitaria (IECS), Buenos Aires, Argentina; ^6^ Harvard T. H. Chan School of Public Health, Boston, MA, United States; ^7^ Faculty of Social Sciences, Tampere University, Tampere, Finland; ^8^ Global Alliance for Chronic Diseases, London, United Kingdom; ^9^ NYU Grossman School of Medicine, New York, NY, United States; ^10^ Juntendo University, Tokyo, Japan; ^11^ University of Antwerp, Antwerp, Belgium; ^12^ University of Illinois Chicago, Chicago, IL, United States; ^13^ La Trobe University, Melbourne, VIC, Australia

**Keywords:** non-communicable diseases, implementation research, training school, capacity strengthening, virtual mode of delivery

## Abstract

**Objective:** To describe the design, delivery and evaluation of the 3rd Global Alliance for Chronic Diseases (GACD) Implementation Science School (ISS), delivered virtually in 2020 for the first time.

**Methods:** Since 2014, GACD has supported the delivery of more than ten Implementation Science Workshops for more than 500 international participants. It has also been conducting an annual ISS since 2018. In this study, we described the design, delivery and evaluation of the third ISS.

**Results:** Forty-six participants from 23 countries in five WHO regions attended the program. The virtual delivery was well-received and found to be efficient in program delivery, networking and for providing collaborative opportunities for trainees from many different countries. The recently developed GACD Implementation Science e-Hub was found to be an instrumental platform to support the program by providing a stand-alone, comprehensive online learning space for knowledge and skill development in implementation research.

**Conclusion:** The delivery of the virtual GACD ISS proved to be feasible, acceptable and effective and offers greater scalability and sustainability as part of a future strategy for capacity strengthening in implementation research globally.

## Introduction

The epidemic of non-communicable diseases (NCDs) is rapidly increasing in most low- and middle-income countries (LMICs) and resource-constrained settings in high-income countries (HCIs) [1]. Many contextual and political factors have hindered the implementation of evidence-based interventions and policies to tackle NCD prevention and control including building robust capacity at national and local levels [2] amongst both researchers and implementers [3]. The challenge of implementing NCD policies and programs in LMICs has led to an increased need for strengthening implementation research capacity [4, 5]. Accordingly, training in implementation research is now a major and critical capacity gap in almost all LMICs [6].

Implementation research capacity strengthening initiatives, although increasing in number, are still limited and are not accessible or affordable for the majority of health program managers, early-career researchers and healthcare professionals in LMICs [7]. Global initiatives include the implementation research training program conducted by the WHO Special Programme for Research and Training in Tropical Diseases (TDR) for many years [8]. This program has applied a training model that focuses on team-based learning, tailored didactic opportunities, learning-by-doing, and mentorship. Though this program primarily focuses on infectious diseases and could be adapted for NCD prevention and control in LMICs, there remains a huge gap in NCD implementation research capacity in LMICs. Among recent NCD research capacity strengthening initiatives in LMICs the US Fogarty-funded ASian Collaboration for Excellence in Non-Communicable Disease (ASCEND) program was implemented and evaluated between 2011 and 2015 in India, Sri Lanka, and Malaysia. This program demonstrated the effectiveness of utilizing blended in-person training with online learning and mentoring of early-career researchers in LMICs to enhance research capacity, performance, and outputs [9].

## GACD’s Investment in Implementation Research in LMICs

The Global Alliance for Chronic disease (GACD) brings together 15 national health and medical research funding agencies with the common mission to reduce the burden of chronic NCDs in LMICs and in populations facing conditions of vulnerability in high-income countries (HICs), by building evidence to inform national and international NCD policies. Collectively, these funding agencies—including the US NIH, UK MRC, Australia’s NHMRC, South Africa MRC—and many other agencies from both high- and middle-income countries—represent over 80% of all public funding for health research in the world. To date, the agencies have invested more than $US250 million in implementation research grants to improve the uptake and scale-up of evidence-based interventions to prevent and control NCDs, including mental health [10].

As part of its mission, GACD has also been supporting a range of implementation research training and capacity strengthening activities in LMICs since 2014, through three main channels: Implementation Science Workshops (ISWs), Implementation Science Schools (ISSs) and most recently, through the development of the GACD Implementation Science e-Hub (https://implementationscience-gacd.org/), which is a free online publicly available resource to advance knowledge and practice in implementation science in relation to NCDs.

GACD has delivered more than ten 2-day ISWs in nine LMICs, reaching more than 500 participants (55% female, 45% male). The majority of participants have been from, or working in, LMICs. In November 2018, GACD launched its inaugural in-person five-day implementation science school (ISS), hosted by the Sao Paulo Research Foundation (FAPESP) in Brazil. This was followed in 2019 by a second ISS in Bangkok hosted by the Health Systems Research Institute, Mahidol University, Thailand. Each of these training schools was supported by senior experts from the GACD Research Network as faculty members and attracted early- and mid-career researchers undertaking their research in LMICs as trainees.

Building on the success of the first two ISS and in order to increase the access to this kind of training, and furthermore, accelerated by the COVID-19 pandemic, the third ISS was conceived and developed as a virtual event over 2 weeks, delivered at the end of November 2020. The advent of this virtually delivered program also provided an opportunity to promote and evaluate a more accessible program delivery format. In this paper, we describe the design and delivery of the virtual ISS as well as findings from the evaluation of its feasibility, acceptability and impact from the participants’ and facilitators’ perspectives. We then discuss strategies for improving the future design and delivery of GACD’s implementation research capacity strengthening programs.

## Methods

### Curriculum and Program Objectives

The GACD ISS (https://www.gacd.org/community/capacity-development/implementation-science-schools-and-workshops) aims to introduce early- and mid-career NCD researchers to the field of implementation research. The learning objectives of the GACD ISS were to: equip participants with the knowledge and skills to identify and address the challenges of implementing NCD policies and interventions in LMICs; support them understand the role and importance of theories, models, and frameworks in implementation research; and introduce them to case studies of implementation research and innovative ways of collaborating and networking in implementation research the future.

### Participants, Faculty Members and Facilitators

#### The Application Process

The ISS was advertised on the GACD website and with email communication to all (700+) members of the GACD Research Network. In addition to completing the ISS application form, applicants were required to submit a structured abstract of a relevant implementation science project, including the identification of a implementation problem or gap. Applicants were also required to submit a letter of support from their mentor or supervisor outlining how the skills they acquired would be applied after this training.

#### The Selection Process

Each application was reviewed independently by two faculty members and the Chief Executive of GACD. Applications were scored based on two major criteria: 1) career development performance and potential; and 2) likely quality and potential impact of the research proposal. The virtual ISS was offered at no-cost to participants. Successful participants were invited to attend a 1-h online workshop, 2 weeks prior to the formal ISS, to discuss the format of the school and technology requirements.

#### ISS Faculty Members and Facilitators

The international faculty members for the ISS were senior implementation science researchers based in *Argentina*, Australia, Finland, Kenya, United Kingdom and United States. In the 2018 and 2019 ISS, face-to-face group sessions were facilitated by the faculty members. For the 2020 virtual ISS, the group sessions were led by facilitators who were selected from previous ISS alumni based on their leadership skills, commitment to the field of implementation science, interpersonal skills and their geographical location. All facilitators attended a 2-h workshop, 2-weeks prior to the formal ISS, to discuss the format of the school, the role of facilitators, available support and technology requirements.

### Program Delivery

The virtual ISS program comprised the following synchronous and asynchronous components: 1) a series of pre-recorded lectures available in advance from the GACD implementation research e-Hub, 2) live online faculty-led plenary sessions, 3) live online facilitator-led small group sessions, 4) recommended readings available from the GACD e-Hub, and 5) access to communication and engagement platforms including Zoom, Padlet and WhatsApp.

#### Pre-Recorded Lectures

International faculty members delivered 19 lectures. These pre-recorded lectures focused on 12 priority topics ranging from an overview of implementation research to building capacity in implementation research in LMICs.

#### Plenary Sessions

Seven 2-h live synchronous plenary sessions were organised (*via* Zoom) over the two-week duration. Participants were expected to watch relevant pre-recorded lectures prior to the corresponding plenary session (refer to Table 1). The relevant faculty members led the discussion on their respective lecture topic and responded to participants’ questions on the topic. Among the plenary sessions there was a showcase of implementation research case studies from China, Africa and India. There was also a panel discussion on “How to make sure that your research findings do not get lost in translation.” The panel included implementation researchers, senior health professionals and policy makers.

**TABLE 1:** List of implementation science lecture topics discussed in each plenary session (Melbourne, Australia, 2020).

Plenary session (PS)	Lecture #	Lecture topics
PS1	Lecture 1a	Overview of implementation science
	Lecture 1b	Implementation science key concepts, models and issues
	Lecture 2	Implementation research questions and designs
PS2	Lecture 3a	Theories, models and frameworks (TMF) in implementation research
	Lecture 3b	TMF—a practical application
PS3	Lecture 4a	Developing a career in implementation research in a global world
	Lecture 4b	Career development story from an implementation researcher from India
	Lecture 5a–5c	Implementation science in LMICs—Case studies from Africa, China and India
PS4	Lecture 6	Building the evidence base for dissemination and implementation research: a population and public health perspective
	Lecture 7a	Interventions and measurements in implementation research part 1
	Lecture 7b	Interventions and measurements in implementation research part 2
	Lecture 8	Implementation research in the real world: how to propose a good topic and get funded
	Lecture 9	Cultural adaptation and constext for program design, implementation and evaluation
PS5	Lecture 10	From surveillance to natural experiments and population monitoring
	Lecture 11	Stakeholder engagement in implementation research
PS6	Lecture 12a	Building capacity for implementation science in LMICs part 1
	Lecture 12b	Building capacity for implementation science in LMICs part 2
	Panel discussion	Panel Discussion: “How to make sure that your research findings do not get lost in translation”
PS7	Closing ceremony	——

#### Facilitated Group Sessions

The participants were divided into eight small groups and each small group was led by a facilitator who had been a previous ISS attendee. Six 2-h small group sessions were organised over the duration of the two-weeks school. The groups discussed and reflect on the learnings gained from pre-recorded lectures and their corresponding plenary session. Each group was asked to identify an implementation problem, formulate an implementation research question, and select a study design and an appropriate theory, model or framework. Each group developed their idea into a consolidated implementation research project proposal over the two-weeks duration and presented it during the final plenary session.

### Implementation Science e-Hub

We developed an Implementation Science e-Hub (https://implementationscience-gacd.org/) [12] that contains the syllabus for the virtual ISS. In addition to pre-recorded lectures and learning materials, it provides self-directed training programs, evidence summaries, interactive learning tools and a specially curated index of international resources. The e-Hub was used as a resource for pre-reading, lectures, and additional reading during the virtual ISS.

### Communication and Engagement Platforms

A Padlet (https://padlet.com/) wall served as a platform for informal communications. Participants were encouraged to introduce themselves and post any questions that they had on a Padlet wall prior to and during the virtual ISS. The faculty and organisers were actively monitoring the Padlet wall and responding to welcome greetings and questions posted by trainees. Furthermore, three WhatsApp groups were created and used for communications related to the ISS. The Zoom chat function was also used for communications during plenary and small group sessions.

### Evaluation Approach

Using the principles of the RE-AIM framework [13] and Kirkpatrick model [14], the program evaluation focused on three key dimensions: Feasibility, acceptability and effectiveness of the virtual ISS. Feasibility was measured by adaptation of the ISS to a virtually delivered program, participants’ attendance and engagement with the Implementation Science e-Hub. Acceptability was assessed using participants perception and reflection of their experience with the virtual ISS. Effectiveness was determined by measuring the achievement of ISS objectives including attainment of learning goals, networking and continuous engagement of participants following completion of the ISS.

### Data Sources and Analysis

We used data from the GACD ISS application form completed by trainees as part of the admission process, and an evaluation survey completed by trainees at the end of the 2-week ISS. The data analysis for this paper was generated using Qualtrics software, Version XM. The e-Hub data analytics were collected over the duration of the ISS. Descriptive statistics and graphs were used to summarize participants’ responses to the survey and thematic analysis was used to summarize data from the open-ended questions.

### Ethics

As per the National Health and Medical Research Council (NHMRC Australia) guidelines, this evaluation is classified under the ‘quality assurance’ category. The data collected and analysed for this evaluation was mainly collected by GACD for identifying areas for improvement for future training, hence did not require a Human Research Ethics Committee review process. However, it was ensured that the questions did not pose any risks and burdens to participants and that anonymity was maintained in analysing any data and the use of an opt-out approach.

## Results

### Participants’ Recruitment and Demographics

**FIGURE 1:**
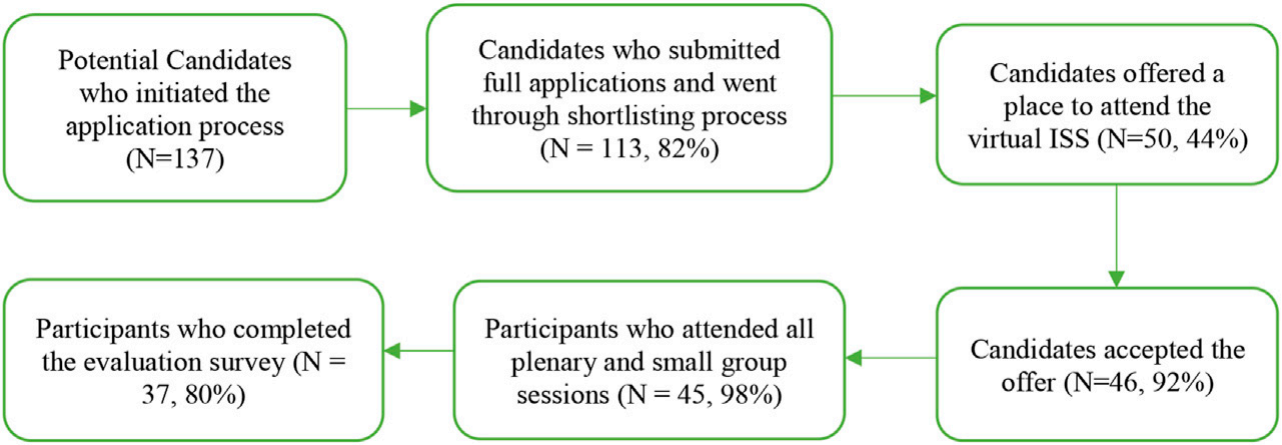
Global Alliance for Chronic Diseases Implementation Science School Participants recruitment (Melbourne, Australia, 2020).

For the virtual ISS conducted in 2020, a total of 137 potential candidates initiated the application process, 113 submitted a full application i.e., provided all the required documentation. Out of these, 50 (44%) candidates were offered a place to attend the virtual ISS, out of which 46 candidates (92%) accepted the offer and attended the ISS. A total of 37 trainees (80%) completed an online evaluation survey at the end of the 2-week ISS (refer to Figure 1).

Of the 46 participants who joined the virtual ISS, 78% were female. Thirty-five (76%) identified themselves as early-career researchers. By residence based on the WHO regions, one-third (30%) participants were from the Southeast Asian region, whereas about one-fourth (22%) were from the African region. About one third (34%) of the participants were associated with existing GACD-funded projects.

### Feasibility of the Virtual Program

Adaptation of the ISS into a virtual delivery format is summarized in the table below (Table 2).

**TABLE 2:** Adaptation from in-person training to virtual training (Melbourne, Australia, 2020).

Aspects of ISS	In-person	Virtual
Duration	Five days	Seven sessions over 2 weeks
# of participants	Global reach but limited to participants who could travel to the host country	Global reach, diverse group of participants from various regions and time zones
Lectures	Face-to-face interaction	Pre-recorded via the Implementation Science e-Hub
Plenary sessions	Live in-person	Live *via* Zoom
Content sharing	PPT slides, handouts (hard copy)	Implementation Science e-Hub
Communication	Live announcements	Live during sessions, and via a WhatsApp group outside sessions
Facilitated learning	Self-learning with other participants	Facilitated by trained facilitators
Technology	Presenters’ device and projector	Zoom, stable internet connection, computer microphone and audio
Networking and social opportunities	Morning/afternoon tea, lunch, social breakout rooms, discussions	Padlet^a^, Zoom chat, small group breakout interactions, and WhatsApp groups
Location, travel, and other logistics	Host institution, training venue, travel, transport, accommodation	Organizing plenary and small group sessions, managing different time zones
Learning support	Live in-person during working hours at the venue	Remote using Padlet and WhatsApp as and when needed

a A cloud-based, real-time collaborative platform available at https://padlet.com/.

#### Participants’ Attendance and Engagement

Despite the differences in time zones and anticipated challenges in internet connectivity in LMICs, the average attendance of 98% was recorded across all plenary and small group sessions. About 84% of participants agreed that the timing of the plenary session was suitable for them, whereas 94% agreed that the timing of the small group sessions was suitable for them. The plenary sessions and small group sessions were interactive, participants actively asked questions from the faculty and facilitators, engaged in the discussion about lecture topics, as well as shared their questions and comments through the Zoom chat feature. There were also active interactions *via* Padlet and WhatsApp groups throughout the program. About 95% of the participants agreed that they were able to connect and communicate with the Faculty, Organizers and other attendees through various online communication platforms. One participant expressed this connectivity in the following words:

“I know that preparing the virtual modality was a huge task, and I really appreciate this effort. This modality gave many of us the opportunity to participate and, although I know the face-to-face modality is better in some senses (for example, networking), the virtual modality allowed us to be actively involved.”

#### Engagement With the Implementation Science e-Hub

The GACD Implementation Science e-Hub that contained all the pre-recorded lectures, was piloted over the duration of the virtual ISS. During the pilot phase, the link to the e-Hub was made available to the training participants only. The analytics data were tracked over the two-week duration of the ISS. In total, there were 1,852 views of the e-Hub, out of which two-thirds (1,182) were specific to the ISS page. The views to the “key resources” page surged in the second week with many attendees actively engaging with the content. All the pre-recorded lectures were viewed multiple times by participants.

On a scale of 0–10 (0 being very hard and 10 very easy to use) for ease of access of e-Hub, trainees and facilitators reported a mean of 8.73 (SD ± 1.83) and 9.00 (SD ± 0.93) respectively. Similarly, the trainees and facilitators scored the usefulness of e-Hub as an implementation science resource at a mean of 8.70 (SD ± 1.78) and 9.14 (SD ± 0.99), respectively. All participants found the pre-recorded lectures easy to access and navigate while 94.6% stated that the training page was well-structured. Almost all trainees (97.3%) were satisfied with their experience of using the e-Hub. In addition, participants provided useful reflections about the ease and accessibility of using e-Hub. Some of the reflections are as follows:

“I like the e-Hub because it provides an avenue to have access to multiple resources at a central location and it has a very easy navigation system with opportunities to select resolutions of videos (for example, 360p 240p or 720hd versions), thus conserving mobile data consumption and network connectivity bandwidth requirements for people in resource-constrained settings.”

“The program presentation was excellent. I like the organization of recorded lectures and reading materials by topics. I found this very useful.”

### Acceptability of the Program

Participants’ perceptions of the virtual ISS. The overall organisation of the GACD ISS3 was rated “very high” by participants (98%) and facilitators (88%). There was a high level of satisfaction among participants of virtual ISS, including its mode of delivery and the topics covered. Participants were asked how satisfied they were with the overall format and the delivery of the ISS, on a scale of 0–10 (0 being extremely dissatisfied and 10 being extremely satisfied). Trainees and facilitators reported a mean of 8.22 (SD ± 1.47) and 8.63 (SD +0.86) respectively.

**FIGURE 2:**
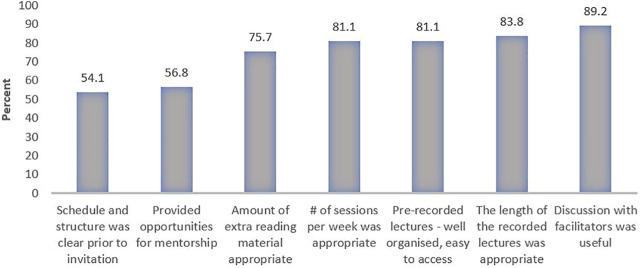
Participants’ reflections on the structure and organization of the virtual Implementation Science School based on the evaluation survey—response scale “strongly agree”/“agree” (Melbourne, Australia, 2020).

Participants were asked about their goals in relation to the virtual ISS. A majority of the participants (90%+) aimed to gain an in-depth understanding of the field of implementation research and expand their learning through case studies and real-world project examples. Most of them also reported that they got a great deal of new information from the virtual ISS. Figure 2 shows some of the main reflections from participants survey.

In addition, participants provided very positive reflections about the structure and organization of the virtual ISS and its specific components. Some of the reflections are as follows:

“I loved the plenary sessions as they put the pre-recorded sessions in context. It was great getting summaries in some areas where I had to watch the pre-recorded sessions after the plenary.”

“I really enjoyed my small group sessions. We discussed many of the tools and methods we learned during plenary and recorded sessions and tried to apply some of them to the group project.”

“I loved the proposal development process. It was great to see the teamwork beyond the time that was allocated for the group sessions.”

“I think this school was organized excellently virtually with a sizable and a diverse group of participants.”

“The virtual program was well designed covering several aspects of Implementation Science.”

### Effectiveness of the Program

#### Increasing Knowledge and Awareness

Most participants (95%) of the virtual ISS stated that the school provided them with clear understanding of implementation research and study designs and that they would be able to apply the knowledge gained from the school in their current job/role in the next six to 12 months. Almost all trainees (92%) agreed that the lectures helped them in understanding the content better and their questions regarding content was answered in a timely manner by program Faculty. Some of the reflections from participants of the virtual ISS in relation to their satisfaction and learnings gained are as follows.

“It was a very inspiring and safe experience overall!”

“The implementation school helped me … to better understand key concepts, designs, and TFM (theories, frameworks and models) and about research. The faculties and facilitators made wonderful work, and the organization in general was so nice.”

“I liked all the pre-recorded lectures. They each have their strengths and highlighted various areas in implementation research. I really liked the session that described the growth in implementation research and that it is a step at a time.”

#### Networking and Mentorship

Most of the participants of the virtual ISS (92%) indicated that the training school provided them with the opportunity to communicate with implementation research experts in the field, and 78% stated that because of attending this training school, they have made professional connections that they aim to leverage in the future to advance their research and/or projects. About 84% of the participants reported that they intend to use the networking and connections from the ISS in their current and future roles, and 78% felt confident that they could use these contacts in the future. Approximately three-quarters of the participants stated that the virtual ISS provided them with opportunities to receive mentorship from faculty and experts in the field of implementation research.

One trainee stated: “I was looking to learn about theories and frameworks, as well as connect with other professionals. That is exactly what happened (in the training).”

### Areas for Future Improvement

Participants suggested some areas of improvement in the virtual ISS including more opportunities to interact with other trainees, more engagement with the faculty, more time for pre-reading and group assignments. Other suggested areas of improvement include the following:

“Since the IS school was in a virtual mode, and we could only interact with a small number of participants from our region, the school did not offer us an opportunity to network widely.

“Would have liked all videos available, one months before plenary. Some were very important for my level of knowledge. Watching in advance a few times, would have helped me.”

“Because of the time difference, the two weeks were likely too short for all the trainees to interact with the Faculty … ”

### Collaboration and Continuous Engagement

Participants of the virtual ISS were keen to continue interactions and collaboration with each other and faculty members in the post-training period. Some participants have proactively stayed in touch within their small groups since the ISS ended in 2020. Additionally, the GACD staff team has been piloting a “Reunion Year” with the alumni. This involves engaging the alumni in collaborative activities, including online reunion events and a closed LinkedIn group to facilitating ongoing networking and learning. The “Reunion Year” content and activities are adapted in real-time in response to alumni feedback and requests. Among the participants of the virtual ISS, 32 alumni signed up to engage with the Reunion Year (70% of all trainees). There were 6 hours of live engagement, across three events that involved five lectures from expert speakers (topics voted for by the alumni). On average 18 alumni attended each event. Furthermore, 26 alumni joined a specialist LinkedIn private group and one asynchronous online journal club delivered over five consecutive days.

## Discussion

Given the challenges associated with the implementation of NCDs interventions and policies in LMICs, the virtual GACD ISS aims to build sustainable implementation research capacity among early- to mid-career NCD researchers in these countries. The virtual ISS was found to be acceptable and feasible in improving knowledge, appreciation, and technical skills to undertake implementation research globally. The facilitator-led group work provided hands on experience of designing a robust implementation research proposal based on the key learnings from the pre-recorded lectures and plenary sessions. The participants had the opportunity to present their collaborative project work to global experts in implementation research and receive feedback in real-time. The school also provided opportunities to participants to establish new connections with other researchers, enabling them to expand their professional networks.

While the total number of trainees who participated in the virtual ISS (N = 46) may seem a small number, limiting the number of attendees to under fifty participants, was a deliberate choice as we aimed for quality over quantity. There was a maximum of 50 participants for this program. Having a smaller number of trainees ensured that our facilitators could provide more focused support to their allocated trainees and trainees had more opportunities to connect with faculty members.

The annual GACD ISS, previously conducted in-person in 2018 and 2019, was successfully adapted for virtual delivery in 2020. Most participants reported that the virtual format continued to provide new knowledge and skills highly applicable to the prevention and control of NCDs in LMICs. The GACD Implementation Science e-Hub piloted during the delivery of the virtual ISS was well-received and widely used and provided a robust platform to support the training programme for the ISS. The e-Hub has subsequently been further enhanced to become a stand-alone, comprehensive open-access online learning space for knowledge and skill development in implementation research, particularly in relation to NCDs. Ongoing enhancements to the e-Hub, beyond its role as a core platform for future ISS, will widen the utility of this resource among the implementation science community, for example offering the ISS materials in the six official UN languages.

Whilst the duration of the virtual ISS was extended (over a two-week period), this digital, innovative, and flexible ISS leveraged digital technology to reach a greater regional diversity of participants to address the high demand in implementation science capacity however, issues of accessibility (mostly through unreliable internet connectivity) for a very small proportion of participants remain a potential aspect of inequity for early career researchers within LMICs.

The virtual model of ISS delivery was found to be more efficient and cost-effective as it did not require participants to physically leave their countries and travel to a central training location**.** Previous in-person training events have required dedicated hosting of the ISS from a GACD Associate Member, significant administrative and logistics effort, commitment from faculty and organisers in taking time out from their work to travel and participate and from trainees to secure both sponsorship (travel and accommodation costs) and time availability (family and work) to travel and attend the 5-day ISS.

In contrast, a virtual ISS and other similar online training events offer the potential to provide learning to a wider geographic scope of trainees that remains engaging but is potentially both more scalable and sustainable and hence can have a longer-term impact than the traditional face-to-face approach. There are some aspects of in-person training that are not easily transferable to online delivery, including the dynamic face-to-face interaction and networking with participants and faculty members. Hence, it may not be appropriate to reconsider the implementation strategies of such events in the GACD capacity strengthening portfolio.

Compared to our previous ASCEND program, the virtual ISS had a better utilization of the online platforms and the e-Hub. However, both programs had a good uptake and effectiveness in terms of improving participants research knowledge and skills. While the ASCEND program evaluation has shown outcomes in terms of research publications and scientific presentation after the training, the GACD ISS is yet to be evaluated for these outcomes. Compared to other Implementation science workshops we have conducted over the last 10 years, the GACD ISS was provided a more comprehensive and well-facilitated implementation research capacity strengthening opportunity for early- to mid-career researchers in the area of NCD prevention and control.

By creating a critical mass of implementation researchers who focus on NCDs, the ISS contributes to effective implementation of impactful NCD research in LMICs by facilitating strong collaborations and partnerships to support GACD investment [15]. In supporting capacity strengthening through its own activities, GACD is keen to catalyse further rippling out of knowledge strengthening and sharing. Implementation science draws on expertise across a range of scientific disciplines, policy and communication skills and engages individuals at multiple career stages. For example, the Brazilian Implementation Science Network is an emerging national activity for implementation research subsequent to an ISS held in Sao Paulo and fosters a platform to enhance collaborative Implementation research in Brazil.

There are some limitations associated with this study. Firstly, most of the data used were self-reported and the possibility of social desirability bias can’t be ruled out. Secondly, this evaluation focused on feasibility, acceptability and immediate indicators of effectiveness. Outcomes of the ISS such as scholarly publications and presentations in scientific conferences by participants are yet to be evaluated. Third, most of the data on participants’ knowledge were collected immediately after the completion of the training. Follow-up studies are needed to assess maintenance of these results. Finally, this study focused on design and delivery of the program. In the longer-term future, further studies are needed to evaluate the impact of the program on the quality of implementation research and on NCD prevention and control in LMICs.

### Conclusion

The delivery of the virtual GACD ISS proved to be feasible, acceptable and efficient and offers greater scalability and sustainability as part of a future strategy for capacity strengthening especially in LMICs. Continuing to broaden the reach of the virtual ISS to a wider range of researchers and healthcare providers working on NCDs would help address the recognised demand in implementation research capacity and will ultimately improve the implementation and effectiveness of NCD interventions and policies worldwide. Further studies are needed to examine the long-term outcomes of the program on research productivity and its ultimate impact on the implementation of NCD policies and interventions in LMICs.

## The Members of the GACD Implementation Science School Facilitators

Beryl Maritim (Academic Model Providing Access to Healthcare (AMPATH), Kenya); Enying Gong (School of Population Medicine and Public Health, China; Academy of Medical Sciences & Peking Union Medical College, Beijing, China); Kavita Singh (Centre for Chronic Disease Control, India); Maria de Lazo Porras (University of Geneva, Peru); Rafael Aiello Bomfim (Federal University of Mato Grosso do Sul, Brazil); Sathish Thirunavukkarasu (McMaster University, Canada).
