# Supplementary material for: Strengthening Capacity for Implementation Research Amid COVID-19 Pandemic: Learnings From the Global Alliance for Chronic Diseases Implementation Science School
Source: Int J Public Health. 2022 Aug 9;67:1604944. doi: 10.3389/ijph.2022.1604944 (PMC9395543; doi:10.3389/ijph.2022.1604944)
Supplement: Supplementary file 1 [file Table1.DOCX]

***Appendix 1. Titles of implementation research projects proposed by small groups*** (Melbourne, Australia, 2020)

| Groups | Title |
| --- | --- |
| A1 | A mixed-method approach to evaluate the HIV Urban Board: A novel digital intervention for adolescents and youth living with HIV to prevent multi-morbidities in low- and middle-income countries |
| A2 | Community Co-designed and led sharing circles: An intervention to prevent and address depression and anxiety in Chiapas, Mexico |
| A3 | Adaptation and integration of PEACE (PTSD Effectiveness, Assessment and Care Component) in the Friendship Bench Program in Zimbabwe |
| A4 | Integrating physical activity for prevention and management of common mental disorders in people with type 2 diabetes in South Africa |
| A5 | Improving Physical activity and reducing sedentary behaviour among Adolescents in Mumbai, India (IMPACT Mumbai) |
| B1 | Community-Health Worker assisted Mobile app-based healthcare program for people with cardiometabolic conditions (COMBAT NCDs) |
| B2 | ReSTORe Healthy China: Reducing Salt intake to Optimum Requirement |
| B3 | Multi-level intervention Package for Prevention and Early detection of Tobacco-related Cancers in Meghalaya, India (MiPE-ToC) |
| B4 | Adherence to Diabetes and Hypertension medication in Odisha, India (ADHmed) |
